# Detailed Dimethylacetal and Fatty Acid Composition of Rumen Content from Lambs Fed Lucerne or Concentrate Supplemented with Soybean Oil

**DOI:** 10.1371/journal.pone.0058386

**Published:** 2013-03-04

**Authors:** Susana P. Alves, José Santos-Silva, Ana R. J. Cabrita, António J. M. Fonseca, Rui J. B. Bessa

**Affiliations:** 1 Unidade de Produção Animal, Instituto Nacional dos Recursos Biológicos (INRB), Vale de Santarém, Portugal; 2 CIISA, Faculdade de Medicina Veterinária (FMV), Universidade Técnica de Lisboa, Lisboa, Portugal; 3 REQUIMTE, Departamento de Geociências, Ambiente e Ordenamento do Território, Faculdade de Ciências, Universidade do Porto, Vairão, Portugal; 4 REQUIMTE, Instituto de Ciências Biomédicas de Abel Salazar (ICBAS), Universidade do Porto, Porto, Portugal; Max Delbrueck Center for Molecular Medicine, Germany

## Abstract

Lipid metabolism in the rumen is responsible for the complex fatty acid profile of rumen outflow compared with the dietary fatty acid composition, contributing to the lipid profile of ruminant products. A method for the detailed dimethylacetal and fatty acid analysis of rumen contents was developed and applied to rumen content collected from lambs fed lucerne or concentrate based diets supplemented with soybean oil. The methodological approach developed consisted on a basic/acid direct transesterification followed by thin-layer chromatography to isolate fatty acid methyl esters from dimethylacetal, oxo- fatty acid and fatty acid dimethylesters. The dimethylacetal composition was quite similar to the fatty acid composition, presenting even-, odd- and branched-chain structures. Total and individual odd- and branched-chain dimethylacetals were mostly affected by basal diet. The presence of 18∶1 dimethylacetals indicates that biohydrogenation intermediates might be incorporated in structural microbial lipids. Moreover, medium-chain fatty acid dimethylesters were identified for the first time in the rumen content despite their concentration being relatively low. The fatty acids containing 18 carbon-chain lengths comprise the majority of the fatty acids present in the rumen content, most of them being biohydrogenation intermediates of 18∶2n**−**6 and 18∶3n**−**3. Additionally, three oxo- fatty acids were identified in rumen samples, and 16-O-18∶0 might be produced during biohydrogenation of the 18∶3n**−**3.

## Introduction

Lipid metabolism in the rumen is characterized by intense lipolysis, fatty acid (FA) biohydrogenation and de novo lipid synthesis by microorganisms [1], determining the lipid composition of ruminant tissues and milk. Indeed, ruminant edible fats are characterized by a high concentration of saturated and *trans*-unsaturated FA, which are associated with deleterious health effects in humans [2]. Nevertheless, some FA produced in the rumen, particularly the 18∶2 *cis*-9,*trans*-11 (a conjugated linoleic acid isomer) have been shown to have important biological properties [3].

Dietary lipids, after ingestion by ruminants, are rapidly hydrolyzed in the rumen by either plant or microbial lipases, which hydrolyze the ester linkages in complex lipids (triglycerides, phospholipids and galactolipids), resulting in the release of FA [1]. After lipolysis, free unsaturated FA might undergo biohydrogenation by the rumen microbiota, which is composed mainly of anaerobic bacteria, ciliate protozoa and anaerobic fungi, although bacteria play the main role in FA biohydrogenation [1]. During the biohydrogenation process, unsaturated FA are isomerized and hydrogenated generating a wide array of isomeric unsaturated FA, mostly with *trans* double bonds, and saturated FA. Both bacteria and protozoa can synthesize and/or incorporate long-chain FA [4]. Indeed, rumen bacterial lipids are characterized by a high proportion of odd- and branched-chain FA (OBCFA), synthesized from propionate and/or branched-chain volatile FA derived from branched-chain aminoacids [5], and also by a high proportion of plasmalogen lipids, containing alk-1-enyl (vinyl) ether chains (herein named dimethylacetals, DMA) [6]. Several ruminal bacteria were also described to convert monounsaturated FA into FA containing hydroxyl or keto (oxo) groups, some authors reporting the hydration of oleic acid (18∶1 *cis*-9) into 10-hydroxystearic, followed by oxidation to 10-ketostearic [7], [8].

Therefore, due to the complexity of the rumen ecosystem and the capacity of rumen microorganisms to perform several FA transformations, the lipid and FA composition of rumen content can be very diverse. Many of the published FA composition of rumen contents do not describe the full variety of products found [9]. Indeed, a high number of FA found in the rumen content makes the analysis very difficult, in part, due to co-elutions during the gas-liquid chromatography (GLC) analysis and the inability of the GLC analysis by itself to characterize all the compounds. Thus, one objective of this work was to improve the methodological approach in order to obtain a detailed FA and DMA composition of rumen contents and to more deeply understand the lipid metabolism in ruminants.

The diet is the major drive of rumen ecosystem, determining the type of microbial consortia established in the rumen and thus affecting the ruminal lipid metabolism, particularly the pattern of biohydrogenation intermediates (BI) generated [1]. However, information on major dietary factors, like basal diet and oil supplementation, on other rumen microbial derived lipids is scarce. We hypothesize that these dietary factors would also induce distinctive patterns on microbial structural lipids in the rumen. Thus, the methodology developed was applied to rumen samples collected during a previous trial [10], with the aim to provide new information about the lipid metabolites found in the rumen of animals fed markedly different diets (concentrate vs. forage with or without soybean oil supplementation), contributing to a deeper insight in rumen lipid metabolism.

## Materials and Methods

### Animals Experiment

The present study was conducted at the Instituto Nacional dos Recursos Biológicos, Unidade de Produção Animal (INRB/INIAV); since it was simply a production trial, where animals were raised according to common production practices, specific ethical approval for this study was not required. However, all studies at the INRB/INIAV fall under the purview of the Direcção-Geral de Veterinária (DGV), and care and sacrifice was done in accordance with the approved recommendations.

Experimental details on animals and diets were published elsewhere [10]. Briefly, Merino Branco lambs were randomly allocated to 4 groups that were fed ad libitum one of 4 pelleted diets: concentrate (C); concentrate plus 10% soybean oil (CO); ground and pelleted lucerne (L); ground and pelleted lucerne plus 10% soybean oil (LO). Lambs stayed on trial for 7 wk and at the end of the trial they were transported to the experimental abattoir of the INRB. Lambs were stunned and slaughtered by exsanguination. After slaughter, the whole rumen content from each lamb was immediately collected and a representative sample was frozen, freeze-dried, milled and stored at −80°C until further analysis.

### Transesterification of Rumen Samples

Freeze-dried rumen samples were transesterified into fatty acid methyl esters (FAME) by using a combined basic followed by acid catalysis adapted from Jenkins [11]. Briefly, 1 ml of toluene and 1 ml of internal standard (19∶0 as free FA, 1 mg/ml) were added to about 250 mg of freeze-dried rumen sample. After the addition of 2 ml of sodium methoxide in methanol (0.5 M), the solution was vortexed and left to react for about 10 minutes at 50°C, after cooling to room temperature 3 ml of 10% HCl solution in methanol was added and the solution was allowed to react for another 15 minutes at 80°C. Once cooled, 6% aqueous potassium carbonate was added in two portions of 2 ml to prevent excessive effervescence, followed by addition of 2 ml of hexane. The solution was then vortexed, centrifuged and the organic layer transferred to another tube. The extraction step with hexane was repeated twice. The final solution was dried over anhydrous sodium sulfate and, after centrifugation, the solvent was collected to another tube and evaporated under a stream of nitrogen at 37°C. The residue was then dissolved in 1 ml of hexane (GC grade).

### Thin-layer Chromatography

**Figure 1:**
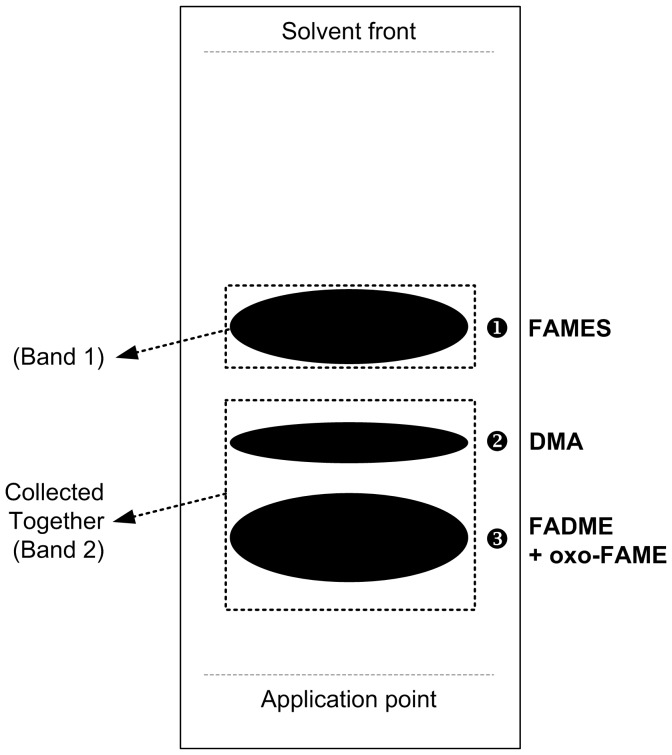
Illustration of a typical TLC plate, indicating the spots corresponding to FAME (1), DMA (2), oxo-FAME and FADME (3), and the bands collected.

An aliquot of 0.5 ml of the dissolved residue obtained after transesterification was evaporated under nitrogen and re-dissolved in about 200 µl of dichloromethane, which was then applied onto the silica-gel plate (10×20). The TLC chamber was saturated beforehand with dichloromethane and the elution was performed using dichloromethane as solvent. Then, the plate was dried and the spots visualized under UV light after spraying with 2′,7′-dichlorofluorescein in methanol. Figure 1 represents a typical TLC plate, showing the spots corresponding to FAME (1 cm band, rf: 0.57±0.05) and the band containing the DMA, oxo-FAME and fatty acid dimethylesters (FADME), which were collected together (3 cm band, rf: 0.29±0.03). Both bands were scraped off and transferred into a new tube. After addition of 1.5 ml of methanol, the fractions were sonicated for about 10 min and then 2 ml of hexane and 1.5 ml of 5% sodium chloride were added. After vortexed and centrifuged (3000 g for 5 min), the organic phase was removed to a clean tube and the aqueous phase re-extracted with another 2 ml of hexane. Finally, the solvent was removed under nitrogen and residues re-dissolved in 1 ml for the FAME fraction and 0.2 ml for the fraction containing the DMA, oxo-FAME and FADME, and transferred to GC-vials and stored at -20°C until analysis. The 19∶0 (50 µg), as methyl ester in hexane, was added as internal standard to the fraction containing the DMA, oxo-FAME and FADME.

### Instrumentation

Samples were analyzed using a gas chromatograph HP6890A (Hewlett-Packard, Avondale, PA, USA), equipped with a flame-ionization detector (GLC-FID) and a CP-Sil 88 capillary column (100 m; 0.25 mm i.d.; 0.20 µm film thickness; Agilent Technologies Inc., Santa Clara, CA, USA). The injector and detector temperatures were 250°C and 280°C, respectively. Initial oven temperature of 100°C was held for 1 min, increased at 50°C/min to 150°C and held for 20 min, increased at 1°C/min to 190°C and held for 5 min, and then increased at 1°C/min to 200°C and held for 35 min. Helium was used as carrier gas at a flow rate of 1 ml/min, and 1 µl of sample was injected. The split ratio used for the analysis of the fraction containing the DMA, oxo-FAME and FADME was 1∶10. For the analysis of the fraction containing the FAME, the typical split ratio was 1∶10. However, in order to get the separation of the 18∶1 *trans*-10 and *trans*-11 isomers, which might coelute in high concentrated samples, a second split ratio of 1∶50 or 1∶100 was used and the amounts of both isomers were calculated from the second run and applied to the area of the first run. For the resolution of the 18∶1 *cis*-9 from both 18∶1 *trans*-13 and *trans*-14 (that co-eluted in our GC-FID conditions), a second temperature program was used. Briefly, the initial temperature column of 70°C was held for 4 min, increased to 110°C at 8°C/min and then increased to 170°C at 5°C/min, held for 10 min, and finally increased to 220°C at a rate of 4°C/min, and maintained for 25 min. Thus, the relative amounts of 18∶1 *cis*-9 and 18∶1 *trans*-13/*trans*-14 were calculated from the second temperature program and applied to the area of the common peak identified in the initial temperature program.

Identification of FAME was also achieved by comparison of the FAME retention times with those of authentic standards (FAME mix 37 components from Supelco Inc., Bellefont, PA, USA, and a Bacterial FAME mix from Matreya LLC, Pleasant Gap, PA, USA). Identification of FAME, DMA, oxo-FAME and FADME was achieved by electron impact (EI) and chemical ionization (CI) mass spectrometry, using acetonitrile as reagent of CI and a Varian Saturn 2200 system (Varian Inc.,Walnut Creek, CA, USA) equipped with the same capillary column and with the oven temperatures used for GLC-FID analysis. The mass spectra conditions were as follow: trap temperature, 150°C; manifold temperature, 45°C; transferline temperature, 220°C; EI ionization energy, 70 eV; scan, 50–650 atomic mass units. The CI parameters, using acetonitrile as reagent, were already described elsewhere [12].

### Statistical Analysis

The experimental unit was the animal, and the effect of basal diet, oil supplementation, and their interaction was analyzed using the MIXED procedure of SAS (SAS Inst. Inc., 2002, Cary, NC), including in the model the group statement for accommodate the variance heterogeneity observed. The significance was declared at *P*<0.05. When interactions between the main effects were significant the means were compared using the least square differences method.

## Results

### Preparation and Separation of FAME, DMA, Oxo-FAME and FADME

**Figure 2:**
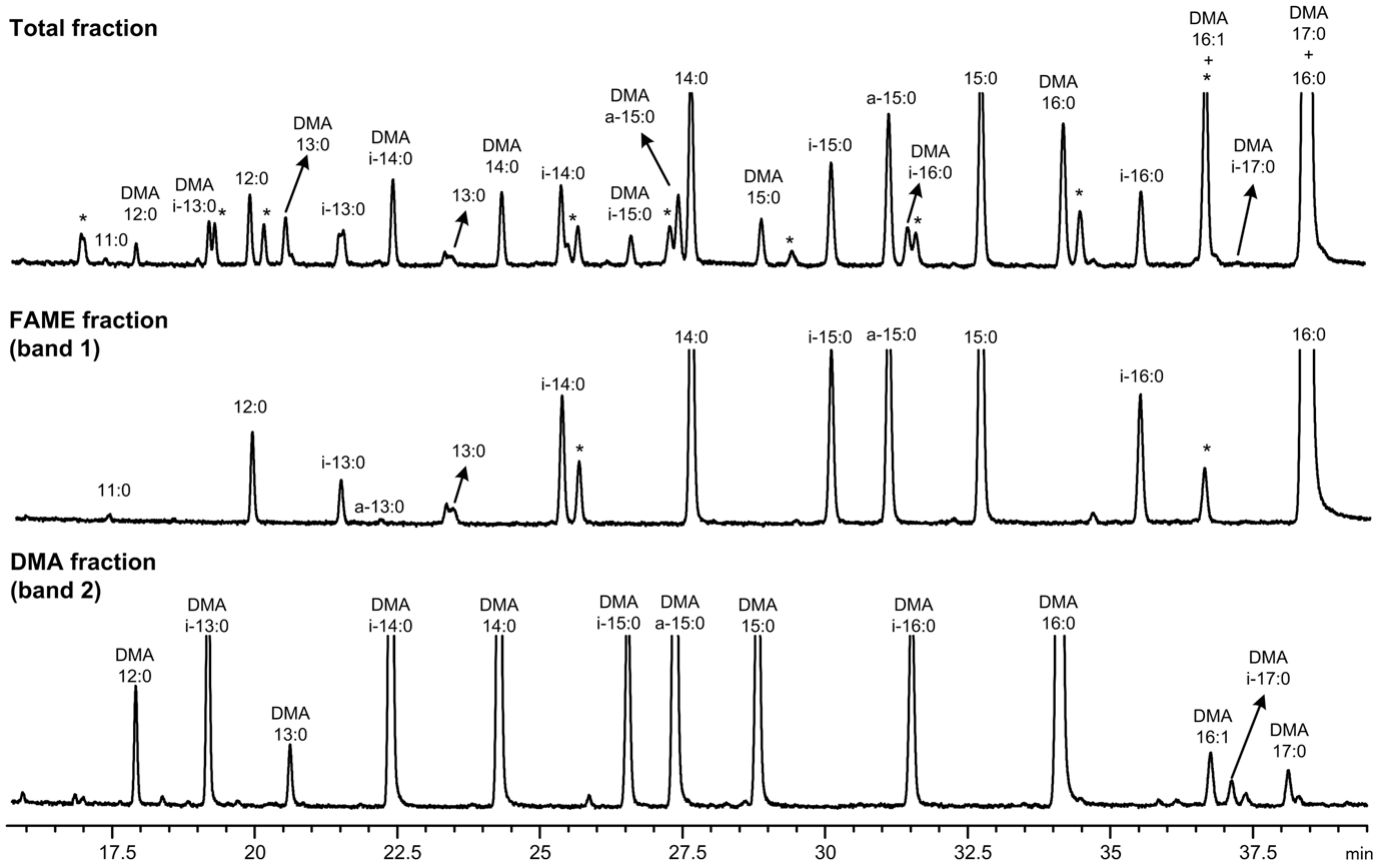
Partial GLC-FID chromatogram (from 16.0 to 39.5 min) of a rumen sample from animals fed lucerne, without TLC fractionation (in the top) and after TLC separation into FAME (in the middle) and DMA fractions (in the bottom). *means unidentified compounds.

**Figure 3:**
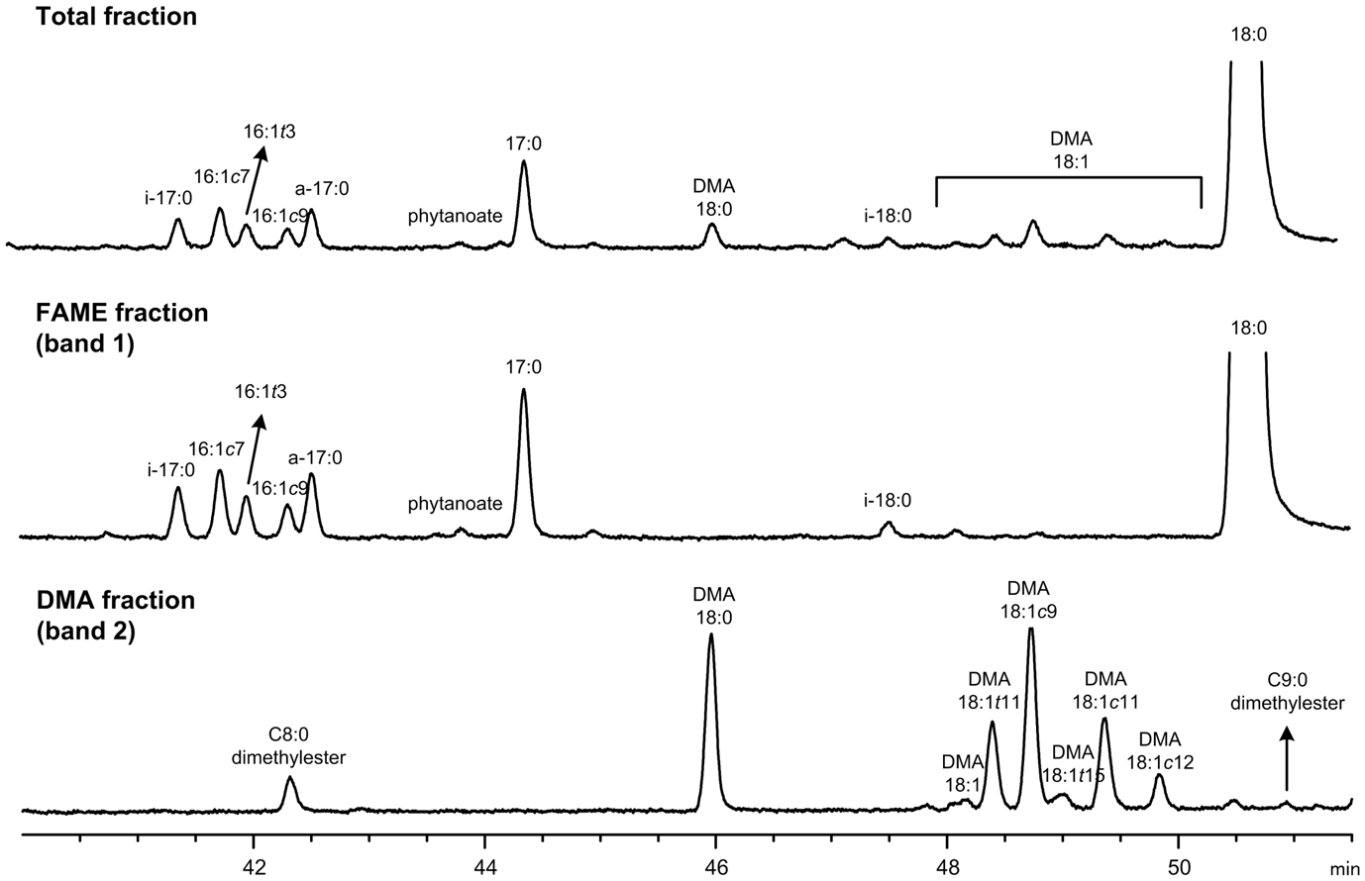
Partial GLC-FID chromatogram (from 41 to 51.5 min) of a rumen sample from animals fed lucerne, without TLC fractionation (in the top) and after TLC separation into FAME (in the middle) and DMA fractions (in the bottom).

**Figure 4:**
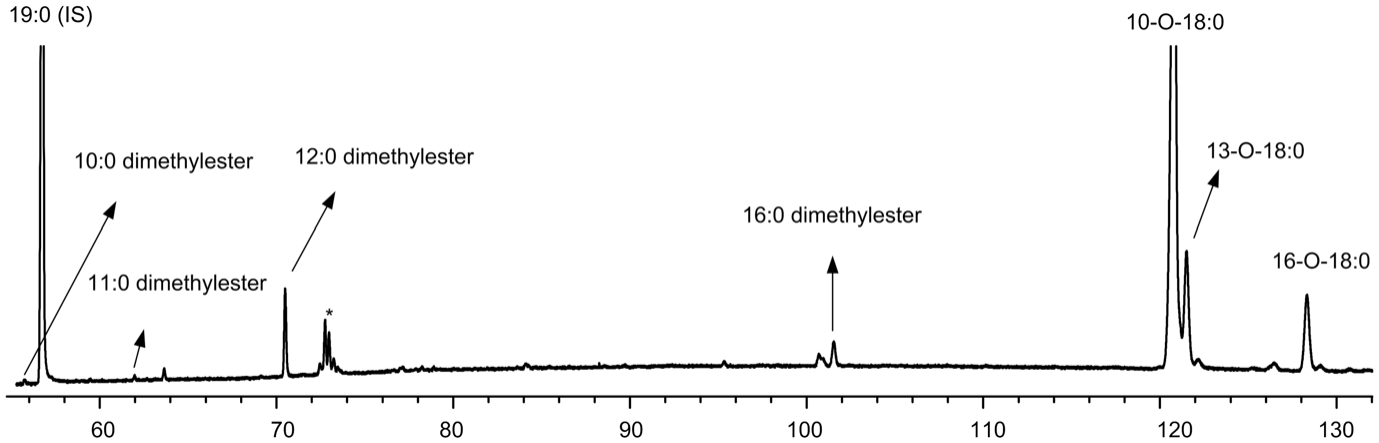
Partial GLC-FID chromatogram (from 55 to 132 min) of the FADME and oxo-FAME region after TLC fractionation of a rumen content sample. *means unidentified compounds.

**Figure 5:**
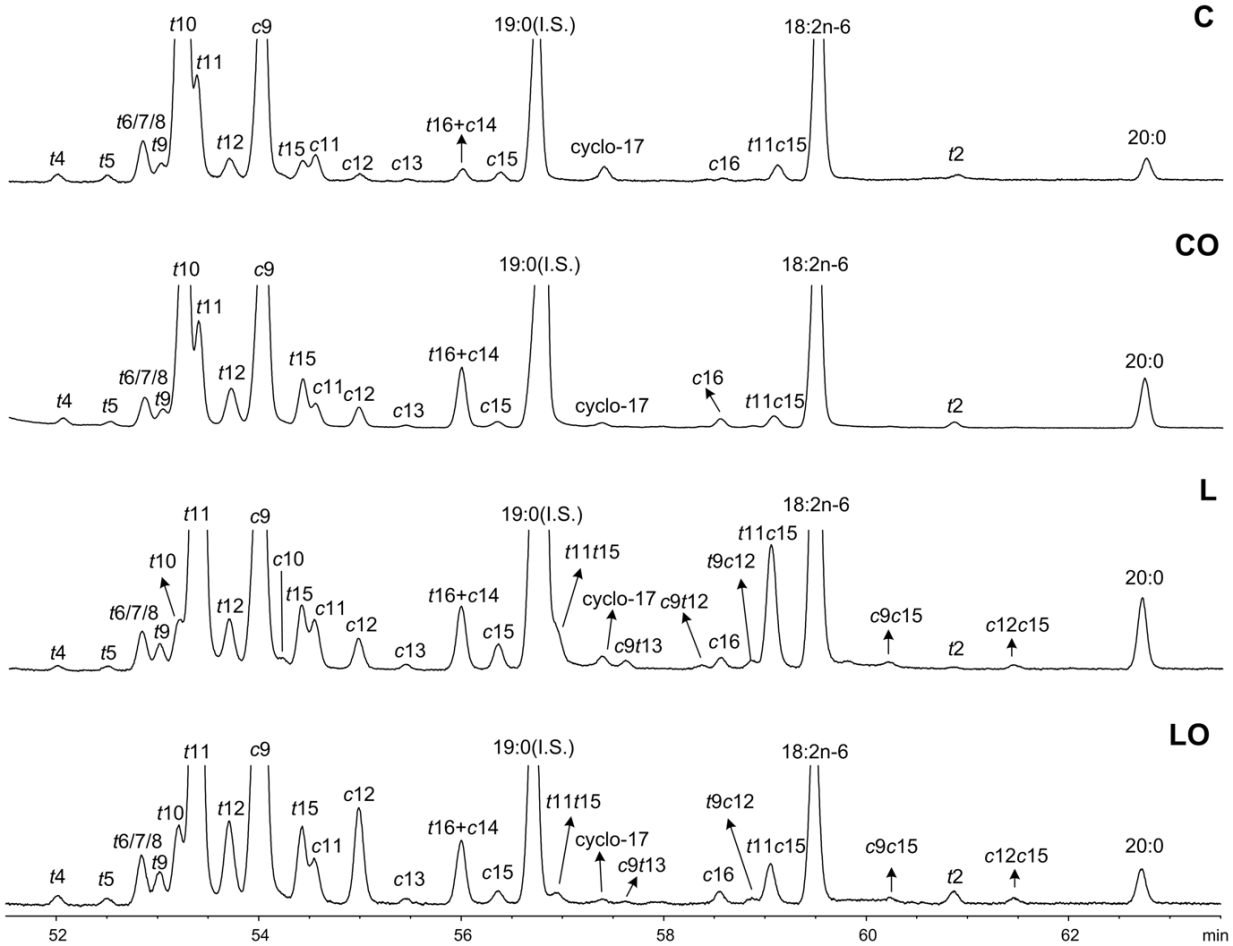
Partial GLC-FID chromatogram of the 18∶1 and 18∶2 FAME region of rumen samples from animals fed: concentrate (C), concentrate plus 10% soybean oil (CO), lucerne (L), and lucerne plus 10% soybean oil (LO).

The basic/acid catalysis of the freeze dried sample generated FAME, DMA, oxo-FAME and FADME. This procedure also methylated the free FA released from soaps in a single step without the need of prior extraction. The TLC fractionation developed allowed the isolation and concentration of FAME from DMA, oxo-FAME and FADME, as shown in Figures 1, 2, 3 and 4. The use of a 100-m long capillary column for the GLC analysis with oven programmed temperature allowed the separation of most of the 18∶1 isomers, as shown in Figure 5. The GLC coupled with mass spectrometry (MS) allowed the confirmatory identification of FAME, DMA, oxo-FAME and FADME.

### Content and Composition of DMA

Table 1 shows the effect of basal diet and oil supplementation in the total DMA concentration and composition (% of total DMA) of the rumen content of lambs fed concentrate or lucerne based diets supplemented (or not) with soybean oil. Twenty four DMA were identified in rumen samples, and the total DMA content was not affected by either basal diet or oil supplementation. Moreover, 5 individual DMA were affected by the interaction of basal diet × oil supplementation, i.e. the i-13∶0, 16∶0, 18∶1 *trans*-11, 18∶1 *trans*-15 and 18∶1 *cis*-9. Independently from the diet, the 16∶0 was the greatest DMA in the rumen content, the highest proportion being found in the rumen of animals fed C diet. The C diet also showed the lowest content of 18∶1 *trans*-11. In contrast, the proportions of i-13∶0 and 18∶1 *trans*-15 were greatest in the rumen contents of animals fed L diet. The oil supplementation had different effects on the two basal diets, with proportions of i-13∶0 increasing in animals fed LO and maintained in animals fed CO diet, compared with unsupplemented ones. Conversely, the soybean oil supplementation showed inversed effects in the proportions of both 16∶0 and 18∶1 *cis*-9 in the rumen content, the proportions maintaining in animals fed the LO diet and tended to decrease in animals fed the CO diet compared with unsupplemented diets, respectively.

**Table 1:** Effect of basal diet (B) and soybean oil (O) in the total DMA content (mg/g DM) and DMA composition (% of total DMA) of rumen content from lambs fed concentrate or lucerne, with or without soybean oil supplementation.

	Diet^1^				*P*-values		
DMA	C	CO	L	LO	B	O	B×O
Total DMA	0.33±0.05	0.42±0.15	0.19±0.02	0.29±0.04	0.108	0.250	0.997
12∶0	1.31±0.24	2.50±0.48	1.34±0.25	2.36±0.37	0.870	0.004	0.815
i-13∶0	0.31^c^ ±0.07	0.40^bc^ ±0.10	2.00^a^ ±0.56	0.67^b^ ±0.09	0.003	0.047	0.024
13∶0	0.73±0.10	0.51±0.07	0.94±0.08	0.90±0.14	0.006	0.223	0.364
i-14∶0	2.67±0.30	3.88±0.63	9.84±0.82	9.73±0.90	<0.001	0.442	0.357
14∶0	4.81±0.43	6.78±0.62	9.92±0.68	11.2±0.96	<0.001	0.030	0.606
i-15∶0	2.11±0.35	1.89±0.29	5.67±0.39	4.47±1.0	<0.001	0.141	0.308
a-15∶0	10.3±2.2	16.4±1.8	11.4±0.9	14.8±1.8	0.862	0.011	0.437
15∶0	4.91±0.60	3.78±0.40	7.22±0.21	5.49±0.38	<0.001	0.002	0.481
i-16∶0	1.14±0.38	2.36±1.01	3.08±0.34	2.66±0.47	0.079	0.522	0.194
16∶0	45.6^a^ ±2.5	35.8^b^ ±2.3	29.0^c^ ±1.5	29.5^bc^ ±2.1	<0.001	0.038	0.026
16∶1	2.01±1.05	1.49±0.35	1.45±0.20	1.23±0.23	0.485	0.524	0.801
i-17∶0	0.57±0.17	0.45±0.06	0.85±0.09	0.63±0.07	0.036	0.117	0.608
a-17∶0	1.84±0.74	1.45±0.47	1.20±0.38	1.46±0.49	0.561	0.900	0.547
17∶0	1.95±0.52	1.21±0.40	0.72±0.09	0.51±0.06	0.007	0.162	0.428
17∶1	1.14±0.16	0.66±0.08	1.16±0.08	1.19±0.17	0.036	0.093	0.054
18∶0	3.32±0.45	2.08±0.18	3.89±0.32	2.02±0.36	0.460	<0.001	0.364
18∶2	0.90±0.13	1.35±0.15	1.34±0.19	1.28±0.20	0.293	0.260	0.139
**18∶1 isomers**							
*trans*-6/−7/−8	0.21±0.03	0.27±0.07	0.26±0.03	0.30±0.05	0.453	0.358	0.776
*trans*-10	2.52±0.62	3.66±1.16	0.43±0.05	0.45±0.04	<0.001	0.388	0.405
*trans*-11	0.80^b^ ±0.14	1.69^ab^ ±0.43	1.79^a^ ±0.15	1.40^a^ ±0.12	0.166	0.326	0.016
*trans*-15	0.34^b^ ±0.04	0.43^b^ ±0.07	0.65^a^ ±0.07	0.36^b^ ±0.02	0.033	0.064	0.002
*cis*-9	8.15^a^ ±1.23	5.36^ab^ ±0.67	3.34^c^ ±0.34	3.91^bc^ ±0.65	0.001	0.173	0.042
*cis*-11	2.51±0.54	5.27±1.06	1.74±0.13	2.02±0.29	0.003	0.020	0.055
*cis*-12	0.41±0.11	1.00±0.23	0.87±0.14	1.93±0.42	0.012	0.003	0.370
**Partial sums**							
∑iso-DMA	6.81^b^ ±0.93	8.78^b^ ±1.41	21.4^a^ ±1.2	18.2^a^ ±1.1	<0.001	0.581	0.033
∑anteiso-DMA	12.2±2.1	17.9±2.1	12.6±0.9	16.0±1.8	0.691	0.016	0.530
∑OBC-DMA^2^	26.6^b^ ±3.3	32.2^b^ ±2.1	42.9^a^ ±1.8	41.1^a^ ±2.3	<0.001	0.452	0.143

a,b,c within a row, means without a common letter differ (*P*<0.05);

1 Diets: C, concentrate; CO, concentrate plus 10% soybean oil; L, pelleted lucerne; LO, pelleted lucerne plus 10% soybean oil;

2 OBC-DMA, odd- and branched-chain DMA. Abbreviations: i, iso; a, anteiso.

Additionally, 11 DMA were affected by basal diet, mostly odd- and branched-chains and a few 18∶1 isomers, whereas 7 DMA were affected by oil supplementation, mostly even-chains, the a-15∶0, 15∶0 and two 18∶1 *cis* isomers. From those affected by basal diet, the proportions of 13∶0, i-14∶0, 14∶0, i-15∶0, 15∶0, i-17∶0, 17∶1 and 18∶1 *cis*-12 were greater in the rumen contents of animals fed lucerne based diets compared with those fed concentrate based diets. The proportion of iso-DMA and total odd- and branched-chain DMA were also greatest in the rumen content of animals fed forage based diets.

The supplementation of both basal diets with soybean oil increased the proportions of several individual DMA, i.e. 12∶0, 14∶0, a-15∶0, 18∶1 *cis*-11 and *cis*-12 and also the sum of anteiso-DMA, and decreased the proportions of 15∶0 and 18∶0.

### Content and Composition of FADME

Several saturated FADME were found in the rumen content of lambs in relatively minor concentration compared to FAME (Table 2) and DMA (Table 1). However, the total concentration of FADME was affected by both basal diet (*P*<0.001) and oil supplementation (*P* = 0.028), showing the greatest concentration in LO diet (0.05 mg/g DM). Regarding the individual FADME, those having lower carbon-chain were affected by oil supplementation (i.e. 9∶0), whereas those with medium carbon-chain were affected by basal diet (i.e. 11∶0, 12∶0, 16∶0), with the exception of 10∶0 that was affected by both factors. The 8∶0 was only detected in concentrate based diets. The 9∶0 and 12∶0 FADME showed the greatest concentration in concentrate and lucerne based diets, respectively.

**Table 2:** Effect of basal diet (B) and soybean oil (O) on the FAME composition (mg/g DM), FADME composition (µg/g DM) and partial sums (mg/g DM) of rumen content from lambs fed concentrate or lucerne, with or without soybean oil supplementation.

	Diet^1^				*P*-values		
	C	CO	L	LO	B	O	B×O
**FAME**							
12∶0	0.10^bc^ ±0.02	0.15^b^ ±0.02	0.07^c^ ±0.01	0.24^a^ ±0.03	0.096	<0.001	0.004
i-13∶0	0.01±0.00	0.03±0.01	0.03±0.00	0.04±0.01	0.030	0.028	0.176
13∶0	0.03^ab^ ±0.00	0.03^ab^ ±0.00	0.02^b^ ±0.00	0.04^a^ ±0.01	0.305	0.096	0.042
i-14∶0	0.08±0.01	0.09±0.04	0.14±0.01	0.15±0.02	0.018	0.574	0.886
14∶0	0.26^b^ ±0.03	0.26^b^ ±0.03	0.29^b^ ±0.01	0.61^a^ ±0.04	<0.001	<0.001	<0.001
i-15∶0	0.13±0.02	0.15±0.02	0.23±0.02	0.29±0.04	<0.001	0.146	0.455
a-15∶0	0.61^a^ ±0.02	0.52^ab^ ±0.08	0.28^c^ ±0.02	0.41^b^ ±0.04	<0.001	0.690	0.025
15∶0	0.27^b^ ±0.04	0.19^b^ ±0.03	0.38^a^ ±0.02	0.51^a^ ±0.08	<0.001	0.657	0.034
i-16∶0	0.10±0.02	0.11±0.05	0.12±0.01	0.16±0.02	0.239	0.266	0.589
16∶0	6.96^b^ ±0.92	12.7^a^ ±1.5	3.89^c^ ±0.20	15.4^a^ ±0.8	0.838	<0.001	0.007
i-17∶0	0.08^ab^ ±0.01	0.06^b^ ±0.01	0.08^ab^ ±0.01	0.12^a^ ±0.02	0.039	0.610	0.025
16∶1*cis*-7	0.13±0.04	0.08±0.02	0.08±0.01	0.10±0.01	0.397	0.542	0.133
16∶1*trans*-3	nd	nd	0.05±0.00	0.06±0.01	−	0.160	−
16∶1*cis*-9	0.05^b^ ±0.01	0.05^ab^ ±0.01	0.04^b^ ±0.00	0.07^a^ ±0.01	0.322	0.031	0.033
a-17∶0	0.17^a^ ±0.03	0.08^bc^ ±0.01	0.07^c^ ±0.01	0.11^ab^ ±0.01	0.068	0.123	0.001
16∶0-3,7,11,15Me^2^	nd	nd	0.02±0.01	0.19±0.03	−	0.001	−
17∶0	0.20^b^ ±0.01	0.26^ab^ ±0.03	0.15^c^ ±0.01	0.31^a^ ±0.01	0.723	<0.001	0.008
i-18∶0	0.04^a^ ±0.00	0.03^b^ ±0.00	0.02^b^ ±0.00	0.05^a^ ±0.01	0.352	0.070	<0.001
17∶1*cis*-9	0.03±0.00	0.04±0.02	0.01±0.00	0.02±0.00	0.212	0.273	0.949
Cyclo-17^3^	0.17^a^ ±0.02	0.09^b^ ±0.01	0.04^c^ ±0.00	0.15^ab^ ±0.03	0.038	0.605	<0.001
18∶0	12.2±1.4	60.4±9.5	5.41±0.28	36.2±3.0	0.005	<0.001	0.095
18∶1*trans* 4	9.89^c^ ±1.93	17.7^b^ ±2.9	2.90^d^ ±0.13	27.8^a^ ±2.2	0.460	<0.001	<0.001
18∶1*cis* 5	3.48^b^ ±0.51	4.81^b^ ±0.66	1.17^c^ ±0.07	9.75^a^ ±0.98	0.050	<0.001	<0.001
18∶2n-6	1.88±0.23	3.48±0.70	1.07±0.05	4.45±0.72	0.879	<0.001	0.095
18∶2 non-conj.^6^	0.13^d^ ±0.02	0.38^c^ ±0.04	0.52^b^ ±0.03	1.46^a^ ±0.09	<0.001	<0.001	<0.001
18∶2 conj. (CLA)^7^	0.46^b^ ±0.14	0.77^b^ ±0.30	0.28^b^ ±0.01	1.95^a^ ±0.20	0.016	<0.001	0.001
18∶3n-3	0.11^c^ ±0.02	0.28^b^ ±0.06	0.82^a^ ±0.05	0.71^a^ ±0.09	<0.001	0.650	0.031
18∶0-oxo^8^	0.18±0.03	0.39±0.07	0.02±0.00	0.31±0.04	0.012	<0.001	0.350
19∶1	0.03^b^ ±0.01	0.04^b^ ±0.01	0.03^b^ ±0.00	0.11^a^ ±0.02	0.005	<0.001	0.004
20∶0	0.21±0.01	0.52±0.07	0.16±0.01	0.60±0.03	0.616	<0.001	0.093
20∶1*cis*-11	0.07^b^ ±0.01	0.09^ab^ ±0.01	0.02^c^ ±0.00	0.11^a^ ±0.01	0.190	<0.001	0.004
21∶0	0.03^c^ ±0.00	0.04^b^ ±0.00	0.03^c^ ±0.00	0.06^a^ ±0.01	0.069	<0.001	0.034
22∶0	0.15±0.01	0.44±0.05	0.17±0.01	0.57±0.02	0.011	<0.001	0.057
23∶0	0.05±0.01	0.08±0.01	0.09±0.00	0.14±0.01	<0.001	<0.001	0.097
24∶0	0.14^c^ ±0.01	0.22^b^ ±0.02	0.17^b^ ±0.01	0.32^a^ ±0.01	<0.001	<0.001	0.040
25∶0	0.03±0.00	0.04±0.01	0.03±0.00	0.05±0.01	0.687	0.014	0.317
26∶0	0.08^b^ ±0.01	0.06^bc^ ±0.01	0.06^c^ ±0.00	0.11^a^ ±0.01	0.034	0.044	<0.001
**FADME**							
8∶0	0.87±0.11	1.42±0.25	nd	Nd	−	0.067	−
9∶0	4.53±1.81	17.0±6.0	1.50±0.47	7.23±2.54	0.069	0.012	0.327
10∶0	2.13±0.39	3.71±0.57	0.92±0.15	2.02±0.34	0.001	0.002	0.543
11∶0	4.12±1.17	4.95±1.17	1.12±0.24	2.04±0.44	0.008	0.400	0.961
12∶0	0.90±0.11	2.32±0.58	28.9±3.3	33.5±4.4	<0.001	0.281	0.563
16∶0	3.61±0.60	4.18±0.38	9.08±1.21	9.20±0.69	<0.001	0.665	0.781
**Partial sums**							
∑FAME	38.4±2.2	105±13	19.0±0.7	103.8±4.1	0.156	<0.001	0.192
∑OBCFA	1.79^b^ ±0.13	1.66^b^ ±0.26	1.69^b^ ±0.08	2.61^a^ ±0.19	0.021	0.029	0.005
∑iso-FA	0.43±0.04	0.46±0.12	0.62±0.04	0.81±0.10	0.002	0.181	0.331
∑anteiso-FA	0.78^a^ ±0.06	0.59^ab^ ±0.09	0.35^c^ ±0.02	0.52^b^ ±0.05	<0.001	0.846	0.004
∑SFA	20.6±1.1	75.3±11.1	10.9±0.5	55.1±3.5	0.017	<0.001	0.379
∑C18^9^	28.3±1.2	88.2±11.1	12.3±0.5	82.8±3.6	0.078	<0.001	0.374
∑BI^10^	11.2±2.02	21.3±3.1	4.20±0.16	34.4±2.4	0.175	<0.001	<0.001
∑FADME	0.02±0.00	0.03±0.01	0.04±0.00	0.05±0.01	<0.001	0.028	0.779

a,b,c,d within a row, means without a common letter differ (*P<*0.05);

1 Diets: C, concentrate; CO, concentrate plus 10% soybean oil; L, pelleted lucerne; LO, pelleted lucerne plus 10% soybean oil;

2 16∶0-3,7,11,15Me, 3,7,11,15-Tetramethylhexadecanoate;

3 cyclo-17, methyl-11-cyclohexylundecanoate;

4 18∶1*trans*, include 18∶1 *trans*-2 and *trans*-4 to *trans*-15;

5 18∶1*cis*; include 18∶1 *cis*-9 to *cis*-16;

6 18∶2non-conj., include *trans*-11,*trans*-15, *cis*-9,*trans*-13, *cis*-9,*trans*-12, *trans*-9,*cis*-12, *trans*-11,*cis*-15, *cis*-9,*cis*-15 and *cis*-12,*cis*-15;

7 18∶2conj. (CLA), conjugated isomers of linoleic acid presented on Table 3;

8 18∶0-oxo, include 10-O-18∶0, 13-O-18∶0 and 16-O-18∶0;

9 C18, includes all FA with 18 carbon chains;

10 BI, biohydrogenation intermediates, include total C18 FA minus 18∶0, 18∶1*cis*-9, 18∶1*cis*-11, 18∶2n-6 and 18∶3n-3. Abbreviations: i, iso; a, anteiso.

### Content and Composition of FAME

Total FAME content, as well as total C18 and SFA concentrations in the rumen content were greatly increased with soybean oil supplementation (*P*<0.001), as shown in Table 2. Total concentration of OBCFA in the rumen content was affected by basal diet × oil supplementation, the greatest concentration being observed in LO diets. Moreover, several individual OBCFA were also affected by basal diet × oil supplementation, i.e. 13∶0, a-15∶0, 15∶0, i-17∶0, a-17∶0, 17∶0, i-18∶0. The general pattern is that oil supplementation increases their concentration in rumen contents of animals fed lucerne based diets and maintain or decreases in animals fed concentrate based diets.

The concentration of FAME with iso structure was more affected by the basal diet than by oil supplementation. Indeed, the concentrations of i-13∶0, i-14∶0 and i-15∶0 as well as total iso-FAME were greatest (*P*<0.05) in the rumen content of animals fed forage based diets (L and LO).

The major individual FAME in rumen content was the 18∶0, the main end product of dietary unsaturated C18 biohydrogenation pathways, reaching the highest concentration in CO diet. The concentrations (mg/g rumen content DM) of major C18 FA are presented in Table 2 and the relative proportions of individual C18 FA (in % of total C18 FA) are presented in Table 3.

**Table 3:** Effect of basal diet (B) and soybean oil (O) on the C18 FA composition (% of total C18 FA) in rumen content from lambs fed concentrate or lucerne, with or without soybean oil supplementation.

	Diet^1^				*P*-values		
FAME	C	CO	L	LO	B	O	B×O
18∶0	44.7^b^ ±6.3	67.3^a^ ±4.9	44.0^b^ ±0.1	44.0^b^ ±3.5	0.011	0.015	0.015
**18∶1 isomers**							
*trans*-2	0.16^b^ ±0.02	0.14^bc^ ±0.02	0.10^c^ ±0.01	0.26^a^ ±0.03	0.227	0.003	<0.001
*trans*-4	0.25^a^ ±0.03	0.12^b^ ±0.01	0.15^b^ ±0.01	0.21^a^ ±0.02	0.847	0.112	<0.001
*trans*-5	0.24^a^ ±0.04	0.12^c^ ±0.01	0.15^bc^ ±0.02	0.18^ab^ ±0.02	0.501	0.087	0.002
*trans*-6/−7/−8	1.30^a^ ±0.12	0.97^ab^ ±0.19	0.88^b^ ±0.05	1.22^a^ ±0.08	0.515	0.939	0.010
*trans*-9	0.64^ab^ ±0.07	0.49^b^ ±0.09	0.56^b^ ±0.03	0.77^a^ ±0.05	0.119	0.657	0.010
*trans*-10	21.6±4.8	13.4±3.5	0.78±0.02	1.40±0.31	<0.001	0.214	0.150
*trans*-11	5.62^c^ ±1.38	1.59^d^ ±0.22	16.6^b^ ±0.56	23.8^a^ ±2.03	<0.001	0.224	<0.001
*trans*-12	1.37^ab^ ±0.16	1.19^b^ ±0.16	1.09^b^ ±0.04	1.67^a^ ±0.12	0.431	0.127	0.006
*trans-13/trans-14*	1.81±0.11	2.07±0.29	2.40±0.19	3.21±0.81	0.062	0.239	0.544
*trans*-15	0.81^b^ ±0.05	1.21^a^ ±0.13	1.27^a^ ±0.07	1.11^a^ ±0.09	0.066	0.196	0.004
*cis*-9	8.95^a^ ±1.23	2.73^c^ ±0.17	5.56^b^ ±0.33	7.34^ab^ ±0.85	0.434	0.008	<0.001
*cis*-10	0.29^a^ ±0.05	0.15^b^ ±0.03	0.23^a^ ±0.02	0.25^a^ ±0.03	0.444	0.092	0.020
*cis*-11	1.09^a^ ±0.07	0.49^c^ ±0.11	0.84^b^ ±0.04	1.00^ab^ ±0.07	0.093	0.008	<0.001
*cis*-12	0.46^c^ ±0.06	0.46^c^ ±0.06	0.69^b^ ±0.07	1.60^a^ ±0.33	<0.001	0.014	0.013
*cis*-13	0.13±0.02	0.09±0.02	0.17±0.01	0.14±0.02	0.028	0.107	0.631
*cis*-14/*tran*s-16	0.69^c^ ±0.07	1.08^b^ ±0.10	1.31^a^ ±0.04	0.98^b^ ±0.09	0.003	0.735	<0.001
*cis*-15	0.25^b^ ±0.03	0.31^b^ ±0.08	0.52^a^ ±0.03	0.24^b^ ±0.02	0.044	0.023	0.001
*cis*-16	0.19^b^ ±0.02	0.21^b^ ±0.02	0.29^a^ ±0.02	0.22^b^ ±0.02	0.014	0.322	0.034
**18∶2 isomers**							
18∶2n-6	6.67±0.78	3.88±0.40	8.70±0.14	5.32±0.83	0.008	<0.001	0.632
*trans*-11,*trans*-15	nd	nd	0.84±0.06	0.37±0.02	−	<0.001	−
*cis*-9,*trans*-13	nd	nd	0.24±0.02	0.11±0.01	−	<0.001	−
*cis*-9,*trans*-12	0.06±0.01	0.02±0.00	0.15±0.03	0.06±0.01	<0.001	0.001	0.062
*trans*-9,*cis*-12	0.09±0.02	0.05±0.01	0.20±0.03	0.14±0.02	<0.001	0.014	0.683
*trans*-11,*cis*-15	0.36^c^ ±0.06	0.34^c^ ±0.05	2.62^a^ ±0.16	0.91^b^ ±0.08	<0.001	<0.001	<0.001
*cis*-9,*cis*-15	nd	0.04±0.01*	0.16^a^ ±0.02	0.10^b^ ±0.02	−	0.015	−
*cis*-12,*cis*-15	nd	0.02±0.01*	0.11±0.02	0.09±0.02	−	0.511	−
**18∶2 conjugated isomers**							
*cis*-9,*trans*-11^2^	0.55±0.21	0.22±0.05	1.21±0.12	1.41±0.19	<0.001	0.662	0.106
*trans*-9,*cis*-11	nd	nd	nd	0.03±0.00	−	−	−
*trans*-10,*cis*-12	0.60±0.20	0.35±0.10	0.19±0.03	0.23±0.06	0.028	0.360	0.208
*trans*-11,*cis*-13	0.08^c^ ±0.01	0.07^c^ ±0.01	0.47^a^ ±0.06	0.25^b^ ±0.02	<0.001	0.002	0.003
*trans*-11,*trans*-13	0.07±0.02	0.02±0.00	0.15±0.02	0.13±0.02	<0.001	0.049	0.479
*trans*-10,*trans*-12^3^	0.46^ab^ ±0.15	0.15^b^ ±0.03	0.25^a^ ±0.03	0.32^a^ ±0.02	0.789	0.137	0.025
**18∶3 isomers**							
18∶3n-3	0.40^c^ ±0.06	0.31^c^ ±0.03	6.66^a^ ±0.18	0.85^b^ ±0.10	<0.001	<0.001	<0.001
*cis*-9,*trans*-11,*cis*-15	nd	0.04±0.01*	0.75^a^ ±0.06	0.19^b^ ±0.06	−	<0.001	−
**Oxo**							
10-O-18∶0	0.58^a^ ±0.10	0.45^ab^ ±0.12	0.04^c^ ±0.00	0.28^b^ ±0.04	<0.001	0.532	0.033
13-O-18∶0	0.03±0.01	0.02±0.00	0.05±0.01	0.05±0.00	0.001	0.942	0.226
16-O-18∶0	nd	0.004±0.001*	0.06^a^ ±0.00	0.04^b^ ±0.00	−	0.015	−
**Partial sums**							
∑Oxo	0.61^a^ ±0.10	0.48^ab^ ±0.12	0.14^c^ ±0.02	0.38^b^ ±0.05	0.002	0.577	0.038
∑CLA	1.61±0.49	0.77±0.17	2.28±0.13	2.36±0.25	0.001	0.207	0.125
∑BI^4^	38.2^ab^ ±5.3	25.3^b^ ±4.5	34.2^b^ ±0.9	41.5^a^ ±2.1	0.107	0.450	0.010

a,b,c,d within a row, means without a common letter differ (*P<*0.05);

1 Diets: C, concentrate; CO, concentrate plus 10% soybean oil; L, pelleted lucerne; LO, pelleted lucerne plus 10% soybean oil.

2 *cis*-9,*trans*-11 coelutes with *trans*-7,*cis*-9 and *trans*-8,*cis*-10;

3 *trans*-10,*trans*-12 might coelute with other *trans,trans* CLA isomers.

4 BI, biohydrogenation intermediates, include total C18 FA minus 18∶0, 18∶1*cis*-9, 18∶1*cis*-11, 18∶2n-6 and 18∶3n−3.

* data not included in the statistical analysis. Abbreviations: i, iso; a, anteiso.

Despite the high concentration of 18∶0 in the rumen of oil supplemented animals (CO and LO diets), in lucerne fed animals, the proportion of 18∶0 in the rumen (% of total C18 FA) remained fairly constant with oil supplementation although, in general, the proportions of BI increased (Table 3). Indeed, BI comprises, collectively, a large fraction of C18 FA, ranging from 25.3% of C18 FA in CO treatment to 41.5% of C18 FA in LO diet.

The BI are produced during the ruminal biohydrogenation of the polyunsaturated FA (PUFA) from the diet, mainly 18∶2n−6 and 18∶3n−3. The concentration of the 18∶2n−6 increased with oil supplementation (*P*<0.001) (Table 2), despite its proportion on total C18 FA being lower in supplemented diets compared with unsupplemented ones (Table 3). The 18∶3n−3 reached 6.7% of total C18 FA in the rumen of animals fed L diet, whereas in concentrate diets it only averaged 0.4% of total C18 FA.

The concentration of the conjugated 18∶2 (CLA) was more affected by the basal diet than by the oil supplementation and reached the greatest concentration in the rumen of animals fed the LO diet. Conversely, the greatest individual CLA in concentrate based diets was the *trans*-10,*cis*-12, which reached 0.6% of total C18 in C diet. The CLA 18∶2 *trans*-10,*trans*-12 was affected by the interaction between basal diet × oil supplementation, the lowest proportion being found in the rumen content of animals fed CO diet.

The proportions of the conjugated 18∶3 *cis*-9,*trans*-11,*cis*-15, and *trans*-11,*cis*-13 CLA were affected by the interaction between basal diet × oil supplementation, and the highest proportions were found in the rumen content of animals fed forage diets, reaching 0.75% (18∶3 *cis*-9,*trans*-11,*cis*-15) and 0.47% (*trans*-11,*cis*-13) on total C18 FA in L diet, whereas in concentrate based diets their proportions were relatively low.

From the non-conjugated and non-methylene interrupted 18∶2 isomers detected in the rumen samples both 18∶2 *trans*-11,*trans*-15 and *cis*-9,*trans*-13 were only detected in lucerne diets, whereas the 18∶2 *cis*-9,*cis*-15 and *cis*-12,*cis*-15 were almost undetected in C diet. Additionally, the proportion of the 18∶2 *trans*-11,*cis*-15 in total C18 FA was higher in forage diets and tended to decrease with oil supplementation. Similarly, oil supplementation decreased the proportions of the 18∶2 *trans*-11,*trans*-15, *cis*-9,*trans*-13, and *cis*-9,*cis*-15 in forage based diets.

Lucerne diets showed the highest (27.8 mg/g DM in LO) and the lowest (2.9 mg/g DM in L) concentration of 18∶1 *trans* isomers in the rumen content. The oil supplementation increased the concentration of 18∶1 *trans* isomers in the rumen content of animals fed concentrate, from 9.89 mg/g DM in C to 17.7 mg/g DM in CO. The concentration of 18∶1 *cis* isomers was also highest in animals fed LO diet (9.75 mg/g DM) and lowest in animals fed L diet (1.17 mg/g DM), and the concentrate diets showed intermediate concentrations, averaging 4.1 mg/g DM.

From all 18∶1 isomers identified in the rumen content only the proportions of *trans*-10, *trans*-13/14 and *cis*-13 were unaffected by the interaction of basal diet × oil supplementation (Table 3). However, the 18∶1 *trans*-10 showed the highest proportion in the rumen content of animals fed concentrate diets (averaging 17.5% of total C18) and the 18∶1 *trans*-11 was the highest 18∶1 isomer in the rumen content of animals fed forage based diets, reaching 23.8% of total C18 FA in LO diet. Concerning the oil supplementation, the general trend is that the inclusion of soybean oil in the diet increases the proportion of individual *trans* 18∶1 isomers and decreases or maintains the proportion of individual *cis* 18∶1 isomers in the rumen content of animals fed lucerne based diets, while tend to have opposite effects in animals fed concentrate based diets.

Other FA were identified in rumen samples, i.e. 10-O-18∶0, 13-O-18∶0 and 16-O-18∶0, namely oxo or keto FA. The concentration of 18∶0-oxo (Table 2) was affected by both basal diet and oil supplementation, the highest concentration being found in the rumen content of animals fed CO diet. The proportions of the individual 10-O-18∶0 (Table 3) was affected by the interaction between basal diet × oil supplementation. The 13-O-18∶0 and 16-O-18∶0 showed the highest proportions in the rumen content of animals fed forage diets, the 16-O-18∶0 being undetected in C diet. Conversely, the 10-O-18∶0 was the highest oxo-FA in the rumen content of animals fed concentrate based diets (Table 3).

## Discussion

### Methodological Approach

Freeze-dried rumen samples from total rumen contents were transesterified using a basic followed by acid catalyst method, adapted from Jenkins [11]. This methodology was chosen because it can directly produce FAME from the intact sample, and save time and solvents compared with methods based on extraction followed by transesterification and because it avoids the isomerization of conjugated FA. Furthermore, due to the large amount of free FA [13] in anionic form and therefore bond to cations, forming salts of calcium or magnesium (soaps) in the rumen, acidic extraction conditions are necessary to release free FA. The common extraction solvent mixtures (chloroform/methanol) applied without acidification could underestimate the amount of FA content in rumen. Moreover, qualitative discrimination could have occurred due to the higher susceptibility of saturated and *trans* monoenes being as soaps form at pH between 6–7 in comparison to other lipids like PUFA [13].

The acidic transesterification method also produced DMA, which are derived from fatty aldehydes, released from plasmalogens. Plasmalogens are a subclass of phospholipids containing a long-chain FA attached at sn-2 position of glycerol and a long-carbon chain attached by vinyl ether bond (alk-1-enyl ether) at sn-1 position. The DMA composition, which reflects the vinyl ether chain of plasmalogens, can be analyzed by GLC using the identical capillary columns used for the analysis of FAME.

The total fractions from the transesterification of freeze-dried rumen contents, apart from FAME and DMA, also contains non-FA material, such as pigments, waxes, sugars, that after introduced into the GLC columns during the chromatographic analysis might either reduce the column life time or interfere with the FAME or DMA analysis. Therefore, a procedure based on TLC was developed in order to clean-up samples for GLC analysis and also to separate FAME from DMA, oxo-FAME and FADME. The TLC procedure was carried out using silica gel plates and dichloromethane as developing solvent, which allowed the complete separation of FAME from the other products (DMA, oxo-FAME and FADME) (Figure 1). Other authors also described the TLC approach to separate FAME from DMA, using instead 1,2-dichloroethane [14] or benzene [15] as developing solvents, although the separation of oxo-FAME and FADME was not demonstrated.

The two separated fractions were analyzed by GLC. Figure 2 and Figure 3 show several partial GLC-FID chromatograms of the FAME and DMA fractions. In each figure, a total fraction (without TLC fractionation) is shown to demonstrate that the TLC step is recommended to clean-up samples and to resolve both DMA and FADME peaks. Therefore the fractionation by TLC allowed not only the purification of samples but also the concentration of the DMA and FADME fraction for a more detailed analysis. Since both oxo and FADME show higher retention times (during the GLC analysis) compared to DMA, they can be analyzed together in the same run of DMA without coelutions (Figure 4), despite the TLC method could be effective in separating all components as illustrated in Figure 1.

Some authors reported that DMA might be degraded in the injection port during GLC analysis [16], [17], resulting in loss of methanol from DMA to form alk-1-enyl methyl ethers (AME, RCH = CH(OCH_3_)). However, these studies were conducted using packed columns for GLC analysis. Santercole et al. [14] reported that the extent of AME formation from DMA is a function of number of variables, including injection temperatures, injection parameters and mode of injection. To overcome this problem some authors converted DMA in other stable derivatives [14], [18]. In our experiment, we did not observe the formation of AME, thus no degradation of DMA occurred during the injection of the sample. More recently other authors have been made an attempt to identify those AME and DMA in muscle of calves [19].

The identification of FAME using a CP-Sil 88 capillary column have been extensively studied by our group [20]. Therefore, a first identification of the FAME in rumen samples was based taking in account their retention times on the capillary column and by comparison with commercial standards. A second confirmation of all FAME was achieved using mass spectrometry under either EI ionization or acetonitrile CI. The 18∶1 *trans*-2 isomer, as far as we know, has never been found in rumen samples, being herein identified based on EI spectrum (Figure S1) and by comparison with published mass spectra (http://lipidlibrary.aocs.org). Taking in account that FA containing double bonds close to carboxyl group, i.e. at C-2 and C-3, show higher retention times in the CP-Sil 88 capillary columns compared with the other FAME [21], the relative retention time of the *trans*-2 during the GLC analysis compared with the others 18∶1 isomers was used to support its identification (Figure 5).

The EI mass spectra of the FADME and oxo-FAME were very conclusive about the number of carbons and the position of the oxo group, respectively, producing diagnostic ions representative of their structure. The mass spectra of the FADME typically presents significant ions which correspond to the loss of two methoxy ions ([M-31]^+^ and [M-64]^+^), indicating the presence of two methyl ester groups (Figure S2). The diagnostic ions in mass spectra of the oxo-FAME are mainly formed by cleavage at both alpha and beta to oxo group and to the loss of methanol from the fragment ions (Figure S3). The acetonitrile CI mass spectra were used to confirm the molecular ions of all FADME and oxo-FAME identified in rumen samples.

Furthermore, the EI mass spectra of the DMA are particularly distinct from the FAME and FADME mass spectra, as they present an abundant base peak at *m/z* 75, correspondent to the McLafferty rearrangement (Figure S4). The first significant ion in the high mass range represents the loss of a methoxy ion [M-31]^+^, and in practice these ions are used to find the molecular weight. Even if the number of carbons can be easily identified, the branched-chain structure (iso and anteiso) or the double bond position in the unsaturated DMA is not easy. Thus, the tentative identification of the branched-chain and unsaturated DMA products was carried out taking into account the retention time and by analogy with the fatty acids.

### Biological Significance

#### Products from microbial origin

The particularity of rumen digesta is that it presents several FA and DMA that are not present, or present in negligible amounts, in the feedstuffs. Some of these compounds are known to result from microbial mediated metabolization of dietary unsaturated FA, the biohydrogenation intermediates, and are discussed bellow. Others are known to be structural lipids of microbial membranes and cell walls, and are de novo synthesized by rumen microbes, participating in the maintenance of optimal membrane fluidity [22]. Plasmalogen lipids are for long known to be present in large amounts in anaerobic bacteria membranes [23] and the presence of the vinyl ether bond gives plasmalogens different physical properties compared to other phospholipids [24]. Indeed, they appear to play a role in regulating the membrane fluidity in several anaerobes [25]. Therefore, rumen microbes might adjust their plasmalogen content and composition in response to different environmental stimuli.

The DMA composition of rumen contents was quite similar to the FAME composition, presenting odd- and branched-chains with carbon lengths between C15 to C18. The DMA composition of representative strains of rumen bacteria has been studied mainly in isolated pure bacteria [6]; [26], and also showed to be relatively rich in odd- and branched-chains. However, as far as we know, there is no information on the effect of the main dietary factors on in vivo rumen DMA content and composition. Dietary factors like basal diet, fat source and supplementation levels can be expected to modify the DMA composition of the microbial cell matter, similarly to what is known to occur to the microbial FA [27].

The lack of effect on the total DMA content coupled with differences on individual DMA proportions among diets suggest that rumen microorganisms could adjusts their plasmalogen composition as a response to different rumen environments, but maintain their total content at same level. Basal diet (forage vs concentrate) affected the proportions of branched-chain DMA, particularly iso-DMA. Similar findings were observed for the iso-FAME, probably indicating shifts in the microbial population. Indeed, different species of rumen bacteria were described to have different patterns of cellular DMA and FA [26]. Differences in forage to concentrate ratio were described to affect the FA composition of the microbial population in the rumen [28] or duodenal content [27], [28] of goats and cows. These studies reported that decreasing the forage:concentrate ratio reduced the iso-FA and increased a-15∶0, probably due to the relative decrease of cellulolytic bacteria over the amylolytic, respectively. Therefore, our results on the DMA and FA composition agree with these findings.

The OBCFA have been described as a potential diagnostic tool of rumen fermentation pattern [5] and as internal microbial markers in the rumen ecosystem [29], thus, we anticipate that the DMA composition derived from plasmalogens could have the same potential. We expect that the DMA potential could be even greater compared with the OBCFA, as DMA are not present in feedstuffs in contrast to odd-FA that can occur, for example, in silages [30].

As discussed in the next section, the *trans* 18∶1 FA are the major BI produced during the microbial biohydrogenation of PUFA in the rumen. Therefore, the presence of a complex isomeric pattern of *trans* 18∶1 DMA in the rumen content indicates that BI can be incorporated in structural microbial lipids, which might reflect a response in the maintenance of the fluidity of cell membranes. The occurrence of unsaturated DMA chains, mainly 16∶1 and 18∶1, have been described in pure [6] and fractionated [23] rumen bacteria, however no discussion about their origin was reported. Additionally, more than 17 alk-1-enyl ethers, including 18∶1 isomers, containing *cis* and *trans* double bonds, were identified in sheep heart [18] and meat [14] plasmalogens. Both studies suggested that the vinyl ether is formed by conversion of the corresponding FA via an alcohol intermediate, following the known aerobic pathway in which the vinyl ether bond is formed at an early stage of the plasmalogen biosynthesis [25]; [31]. However, in anaerobic microorganisms the plasmalogen biosynthesis was demonstrated to be different [25]; [32]. The genes for their biosynthesis have not been discovered yet, although the hypothesis taken by a few authors is that the vinyl ether of plasmalogens in anaerobes is formed at a late stage of the phospholipids biosynthesis [32]. The studies from Goldfine group suggested two hypotheses for the formation of the vinyl ether in plasmalogens [32], in the first, the vinyl ether is formed by direct conversion of the FA while attached to the phospholipid. In the second hypothesis, the 1-linked FA in phospholipids is removed and replaced by a vinyl ether. This ether is probably derived from a FA present in the medium, through the formation of an aldehyde intermediate.

Residual amounts of medium-chain FADME were detected in all rumen samples irrespective of the diets. These FADME were probably methylated from the corresponding dicarboxylic fatty acids (DFA) during the transesterification procedure. As far as we know, the occurrence of DFA has never been described in the rumen content. Nevertheless, they have been found in aerobic bacteria [33], cyanobacteria [34] and yeast [35]. In plants, DFA are components of the natural protective polymers cutin and suberin. Particularly, long-chain DFA with vicinal dimethyl branching were found in the past as major components of the lipids of the Butyrivibrio spp. [36], however these particular long-chain DFA were not detected in our samples.

Medium-chain DFA might be derived from medium and long-chain saturated and unsaturated FA by microbial ω-oxidation [33], [35]. Some studies using aerobic bacteria reported the ω-oxidation of FA to the corresponding DFA, with the formation of the ω-hydroxy acid intermediate [33]. Moreover, in vitro experiments, with *Candida cloacae* cells, grown in the presence of saturated FA demonstrated the formation of the corresponding DFA [35]. These authors also showed that some unsaturated FA with longer chains are preferentially oxidized compared with their homologous saturated, suggesting that the selective ω-oxidation of FA and accumulation of DFA may reflect adaptations of the membrane lipids [35]. Moreover, Passi et al. [37] demonstrated that upon lipid peroxidation, PUFA originate specific DFA which may be regarded as a distinctive feature of the particular positions of the double bonds in PUFA, in which the most abundant DFA is related to the first double bond in the molecule [37].

Hence, and according to the literature, DFA might be derived from either saturated or unsaturated FA through oxygen dependant mechanisms. In ruminal contents, despite the abundance of anaerobic microorganisms, it also contains facultative aerobic and microaerophiles bacteria [38]. From here, we can anticipate that the aerobic microbial population might be involved in the oxidation of FA, probably with a preference for the unsaturated FA, leading to the formation of DFA. However, more work should be done to understand their occurrence in the rumen.

#### C18 fatty acids and biohydrogenation intermediates

Our data did not allow a quantitative evaluation of the biohydrogenation of C18 unsaturated FA in the rumen. However, the C18 FA distribution, particularly of the BI, in the rumen may allow us to identify BI pattern shifts related to dietary conditions. The C18 FA comprise the majority of FA present in the rumen content, this reflects the fact that dietary lipids are also mostly unsaturated C18 FA [10]. Rumen samples from lambs fed concentrate based diets supplemented with soybean oil presented the extreme values regarding the 18∶0 and BI proportions. The high percentage of 18∶0 (Table 3) and the low of BI observed in CO diet suggest that the activity of bacteria able to produce stearic was not compromised by either concentrate based diets or soybean oil supplementation. This is confirmed by the fact that the same pattern is also present in the abomasal digesta from these animals (Figure S5). However, these results do not follow the general concept that ruminants fed high concentrate diets with PUFA overload have a less complete biohydrogenation [39] due to either lower rumen pH, affecting negatively the lipolysis [40], or to toxic effect of dietary PUFA to biohydrogenating bacteria producing stearic acid [41]. In our experiment, the rumen pH was not measured, thus associations between pH and high concentrations of 18∶0 and low of BI in CO diet cannot be performed. But the absence of depressing effects of the PUFA overload (10% of soybean oil dietary inclusion) on the 18∶0 production is clear, suggesting that, in vivo, the C18 PUFA did not exert a relevant toxic effect on biohydrogenating bacteria producing stearic acid.

Despite the accumulation of 18∶0 in the rumen content, the biohydrogenation of dietary unsaturated FA, mainly 18∶2n−6 and 18∶3n−3, has been associated with the occurrence of a wide array of BI in the rumen and with a decrease of the parent unsaturated FA [1]. The 18∶1 *trans*-10 and *trans*-11 were the highest BI accumulated in the rumen content of animals fed concentrate and forage diets, averaging 51% and 53% of total BI, respectively. Indeed, the 18∶1 *trans*-11 is a well known BI of the 18∶2n−6 and 18∶3n−3 biohydrogenation pathways [1]. However, when animals are fed concentrate based diets the rumen biohydrogenation pathway shift to *trans*-10 accumulation [42]. The biologic properties of 18∶1 *trans*-10 are not completely known, although it has been associated with milk fat depression syndrome in dairy cows [43]. Conversely, increasing the 18∶1 *trans*-11 rumen outflow is desirable as it acts as a substrate for the Δ9 desaturase in the animal tissues resulting in the formation of the *cis*-9,*trans*-11 CLA [42], which has been shown to have potential health benefits in humans [3]. However, no endogenous CLA (*trans*-10,*cis*-12) synthesis is possible from 18∶1 *trans*-10, as discussed elsewhere [10].

The interactions between oil supplementation and basal diet observed in the rumen for 18∶1 *trans*-11 were consistent with what was observed in the meat of these animals [10]. The soybean oil was highly effective in order to obtain lamb meat enriched with 18∶1 *trans*-11 and *cis*-9,*trans*-11 CLA when supplemented lucerne but not concentrate [10].

The distinct proportions of the 18∶1 *trans*-10 and *trans*-11 in the rumen content, as well as the separation of the others C18 FA, among the four diets is illustrated in Figure 5. It can be seen that apart from the 18∶1 *trans*-10 and *trans*-11, several others 18∶1 isomers were produced in the rumen which can be further accumulated in milk and meat tissues. Indeed, the putative metabolic pathways of 18∶2n−6 and 18∶3n−3 predicts each, 10 octadecenoic FA isomers, ranging from *trans*-6 to *cis*-16 [39], [43]. Recently, during *in vitro* incubations with labeled 18∶1 *trans*-11, Laverroux et al. [9] described that the *trans*-11 could be isomerized to *cis* and *trans* 18∶1 isomers ranging from Δ6 to Δ16, with more isomerization in forage diets than in concentrate diets. The occurrence of the *trans*-4 and *trans*-5 has been barely reported in the literature, although a few authors have described their occurrence in the rumen [44], omasal content [45], meat [46] and milk [47]. However, as far as we know, the occurrence of 18∶1 *trans*-2 in the rumen content has not been reported.

Several conjugated FA were detected in the rumen content. The *trans*-9,*cis*-11 CLA was only detected in the rumen content of animals fed lucerne supplemented with soybean oil, and the 18∶3 *cis*-9,*trans*-11,*cis*-15 was not detected in the rumen content of animals fed C diet. From some other studies, 18∶3 *cis*-9,*trans*-11,*cis*-15 is a well known BI of the 18∶3n−3 [1]. Also, the *trans*-11,*cis*-13 CLA was described to be an intermediate of the 18∶3n−3 hydrogenation by the *B. fibrisolvens* [48]. This explains the highest proportions of these conjugated BI in the rumen content of animals fed lucerne based diets. However, as soybean oil is richer in 18∶2n−6 than in 18∶3n−3, the BI expected to be derived exclusively from 18∶3n−3 biohydrogenation were diluted by the other BI. Similar effect was observed for the non-methylene interrupted 18∶2 isomers in the rumen content from animals fed lucerne based diets. The 18∶2 *trans*-11,*cis*-15, is a well known BI, resulting from hydrogenation of the *cis*-9 double from the 18∶3 *cis*-9,*trans*-11,*cis*-15 [1]. Moreover, the 18∶2 *cis*-9,*trans*-13 was suggested to be formed by ruminal biohydrogenation of 18∶3n−3 through the reduction of isorumelenic (18∶3 *cis*-9,*trans*-13,*cis*-15) [49]. However, this later octadecatrienoic isomer was not detected in our samples. The 18∶2 *trans*-11,*trans*-15 have been previously reported in milk [50], omasal digesta [45], rumen fluid [51], lamb meat [52] and beef [53], and it is also associated with dietary intakes of 18∶3n−3. The occurrence of the 18∶2 *cis*-9,*cis*-15 and *cis*-12,*cis*-15 in lamb meat was already described by our group [46], furthermore these BI were proposed to be products of the ruminal biohydrogenation of the 18∶3n−3, resulting from the reduction of the *cis*-12 or *cis*-9 double bonds, respectively.

The 18∶2 *cis*-9,*trans*-12 and *trans*-9,*cis*-12 are geometric isomers of the 18∶2n−6, which might be produced from the biohydrogenation of the 18∶2n−6 according to the findings of Jouany et al [44] based on *in vitro* ruminal incubations. However, we did not found an increase of these isomers with soybean oil supplementation.

Particularly some other BI having 18-carbon chain lengths and an oxo group were found in the rumen content. A few early reports showed the occurrence of trace amounts of oxo-FA in milk fat [54], cheese [55] and rumen [56]. In the rumen fluid of cows fed hay, Katz and Keeney [56] isolated nine oxo-18∶0 FA, from these the 16-O-18∶0 was the most abundant. In our study, we detected three oxo-18∶0 FA in the rumen content, being the 16-O-18∶0 the most abundant in lucerne diet. As lucerne is rich in 18∶3n−3, we hypothesize that the 16-O-18∶0 might be produced during the biohydrogenation of 18∶3n−3, particularly by hydration of double bond at C-15, followed by oxidation to produce the oxo group at C-16. This hypothesis also implies the hydrogenation of the double bonds at C-9 and C-12. The inclusion of soybean oil in lucerne diets increased the proportions of 10-O-18∶0 in the rumen content, which was also the most abundant oxo-FA in the rumen of lambs fed concentrate based diets. Indeed, the 10-O-18∶0 was described to be formed from biohydrogenation of the 18∶1*cis*-9, through a hydroxyl intermediate (10-OH-18∶0) [7], [8], [43]. Concentrate diets, as well as soybean oil were described to contain higher proportions of 18∶1*cis*-9 compared with lucerne diets [10], this might explain the highest proportions of 10-O-18∶0 in the rumen of animals fed C, CO and LO diets.

The 13-O-18∶0 was hypothesized to be formed from the biohydrogenation of 18∶2n−6, through conversion to the *cis*-9,OH-13 18:1, reduction to OH-13-18∶1, followed by oxidation to the 13-O-18∶1 [57]. Therefore, the 13-O-18∶0 reached the highest concentration in the rumen content of animals fed soybean oil supplemented diets, which are rich in 18∶2n−6. The incorporation of the oxo-FA into meat and milk should be further explored, as well as their biological and physiological consequences in human’s consumption.

### Conclusions

The detailed DMA, FAME, FADME and oxo-FAME composition of the rumen can be determined using the methodological approach proposed, which is based on a direct basic/acid transesterification of freeze-dried rumen samples followed by TLC fractionation and analysis by GLC.

Different patterns of DMA were observed between basal diets and with oil supplementation, although the basal diet was the factor that contributed the most to the differences in the DMA composition, suggesting the presence of different microbial populations in forage based diets compared with concentrate based diets. However, despite the differences in the DMA composition, the total DMA concentration was not affected by either basal diet or oil supplementation, suggesting that apparently the plasmalogen levels in rumen microorganisms remain at constant levels. More work should be done to study the potential of using DMA as a diagnostic tool of fermentation patterns or as internal markers in the rumen ecosystems. Moreover, the incorporation of BI into plasmalogens should also be considered in future research.

The detection of the FADME in the rumen content may indicate oxidation of FA by aerobic microorganisms, although more work should be done to understand their occurrence in the rumen content.

The 18∶1 composition in the rumen content confirmed the shift in the normal biohydrogenation pathway when ruminants are fed high concentrate diets through the presence of higher levels of 18∶1 *trans*-10 in concentrate based diets and 18∶1 *trans*-11 in forage based diets. However, the common findings that concentrate plus PUFA overload inhibits the growth of bacteria able to hydrogenate 18∶1 isomers to stearic acid were not confirmed.

Furthermore, the complete characterization of the BI in the rumen content allowed the identification of 3 FA with oxo groups. We hypothesize that the 16-O-18∶0 might be produced during the microbial biohydrogenation of the 18∶3n−3, as its content was higher in rumen content of animals fed lucerne.

## Supporting Information

Figure S1 **Electron impact ionization mass spectrum of the 18∶1** ***trans*** **-2 FA detected in rumen samples, showing the diagnostic ion at** ***m/z*** **113 and the characteristic fragments correspondent to the M^+^, [M-32]^+^ and [M-74]^+^.**
(TIF)

Figure S2 **Electron impact ionization mass spectra of the FADME identified in rumen samples, showing the characteristic fragments [MH]^+^, [M-31]^+^, [M-64]^+^, [M-73]^+^ and [M-105]^+^.**
(TIF)

Figure S3 **Electron impact ionization mass spectra of the oxo-FAME identified in rumen samples, showing the characteristic fragments formed by cleavage both alpha and beta to the oxo group.**
(TIF)

Figure S4 **Electron impact ionization mass spectra of the DMA identified in rumen samples, showing the characteristic ion at** ***m/z*** **75 and the common fragments [M-31]^+^.**
(TIF)

Figure S5 **Concentrations of 18∶0, 18∶2n−6 and total 18∶1** ***trans*** **and 18∶1** ***cis*** **in the abomasal digesta from animals fed: concentrate (C), concentrate plus 10% soybean oil (CO), lucerne (L), and lucerne plus 10% soybean oil (LO).**
(TIF)
