# Supplementary figures and images for: Detailed Dimethylacetal and Fatty Acid Composition of Rumen Content from Lambs Fed Lucerne or Concentrate Supplemented with Soybean Oil
Source: PLoS One. 2013 Mar 4;8(3):e58386. doi: 10.1371/journal.pone.0058386 (PMC3587585; doi:10.1371/journal.pone.0058386)

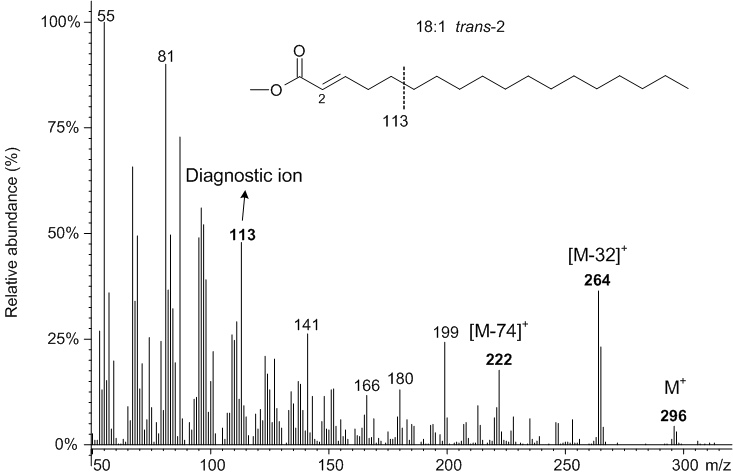

Supplement: Figure S1 — Electron impact ionization mass spectrum of the 18∶1 trans -2 FA detected in rumen samples, showing the diagnostic ion at m/z 113 and the characteristic fragments correspondent to the M+, [M-32]+ and [M-74]+. (TIF) [file pone.0058386.s001.tif]

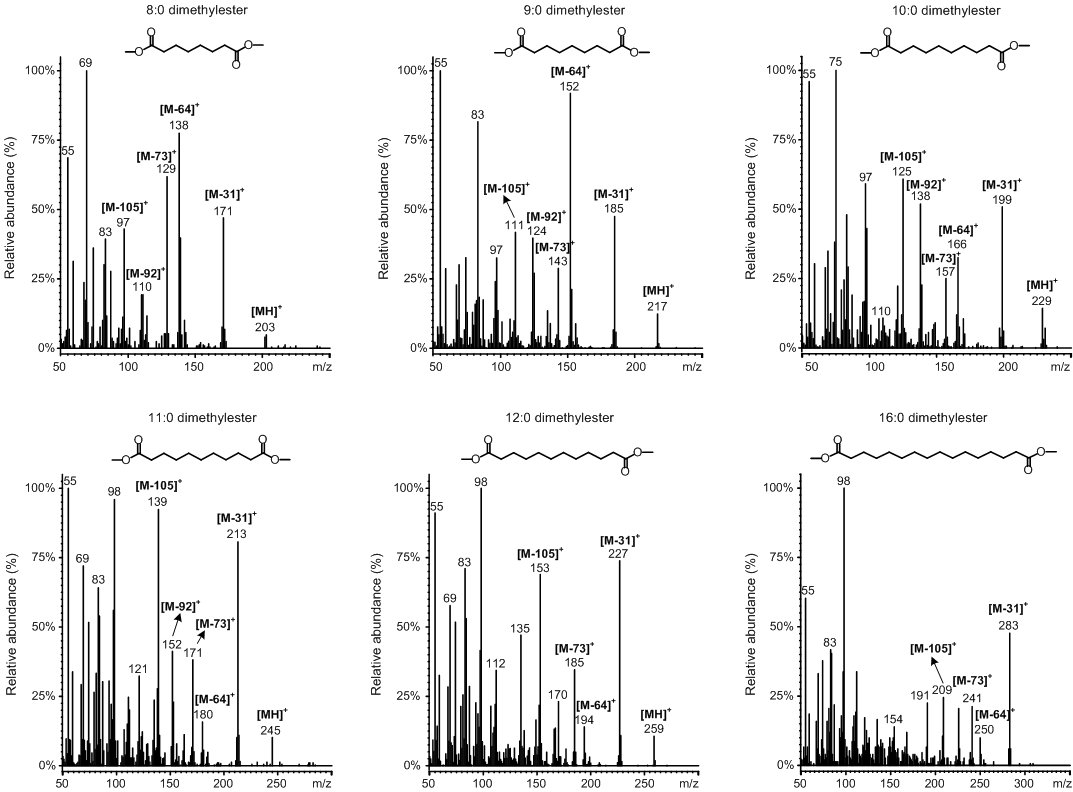

Supplement: Figure S2 — Electron impact ionization mass spectra of the FADME identified in rumen samples, showing the characteristic fragments [MH]+, [M-31]+, [M-64]+, [M-73]+ and [M-105]+. (TIF) [file pone.0058386.s002.tif]

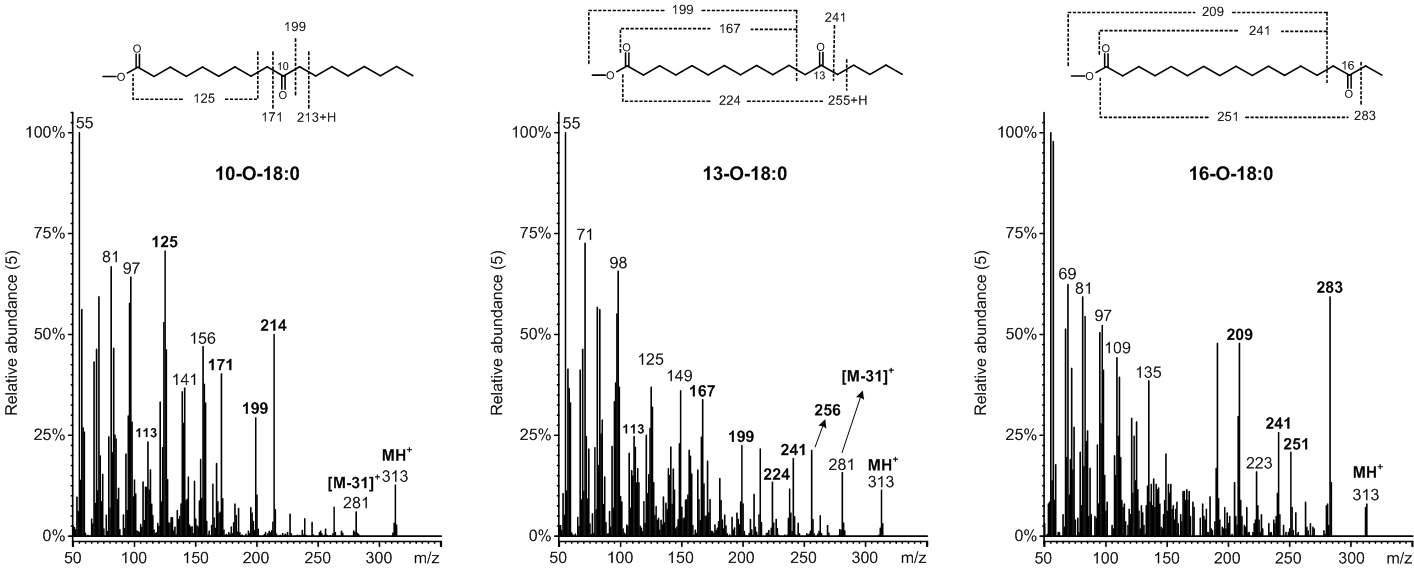

Supplement: Figure S3 — Electron impact ionization mass spectra of the oxo-FAME identified in rumen samples, showing the characteristic fragments formed by cleavage both alpha and beta to the oxo group. (TIF) [file pone.0058386.s003.tif]

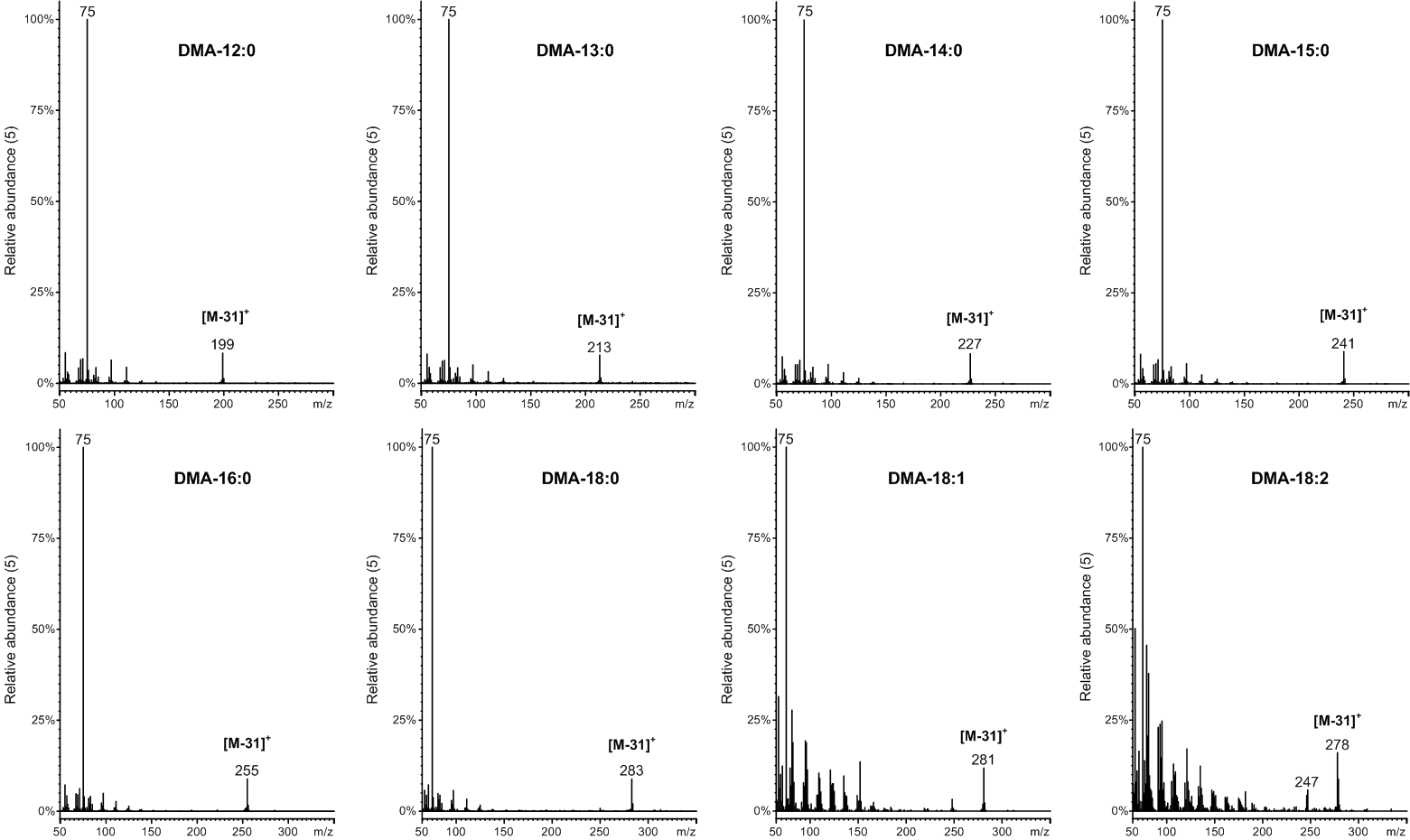

Supplement: Figure S4 — Electron impact ionization mass spectra of the DMA identified in rumen samples, showing the characteristic ion at m/z 75 and the common fragments [M-31]+. (TIF) [file pone.0058386.s004.tif]

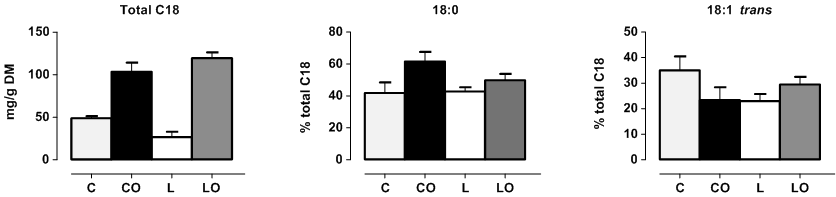

Supplement: Figure S5 — Concentrations of 18∶0, 18∶2n−6 and total 18∶1 trans and 18∶1 cis in the abomasal digesta from animals fed: concentrate (C), concentrate plus 10% soybean oil (CO), lucerne (L), and lucerne plus 10% soybean oil (LO). (TIF) [file pone.0058386.s005.tif]
